# Saffron extract interferes with lipopolysaccharide-induced brain activation of the kynurenine pathway and impairment of monoamine neurotransmission in mice

**DOI:** 10.3389/fnut.2023.1267839

**Published:** 2023-10-05

**Authors:** Camille Monchaux de Oliveira, Jennifer Morael, Alexandrine Guille, Camille Amadieu, Sylvie Vancassel, David Gaudout, Lucile Capuron, Line Pourtau, Nathalie Castanon

**Affiliations:** ^1^INRAE, NutriNeuro, UMR 1286, Bordeaux University, Bordeaux IPB, Bordeaux, France; ^2^Activ’Inside, Beychac-et-Caillau, France

**Keywords:** nutritional interventions, saffron extract, neuroinflammation, lipopolysaccharide, sickness behavior, kynurenine pathway, serotonergic neurotransmission, dopaminergic neurotransmission

## Abstract

**Background:**

Although activation of inflammatory processes is essential to fight infections, its prolonged impact on brain function is well known to contribute to the pathophysiology of many medical conditions, including neuropsychiatric disorders. Therefore, identifying novel strategies to selectively counter the harmful effects of neuroinflammation appears as a major health concern. In that context, this study aimed to test the relevance of a nutritional intervention with saffron, a spice known for centuries for its beneficial effect on health.

**Methods:**

For this purpose, the impact of an acute oral administration of a standardized saffron extract, which was previously shown to display neuromodulatory properties and reduce depressive-like behavior, was measured in mice challenged with lipopolysaccharide (LPS, 830 μg/kg, ip).

**Results:**

Pretreatment with saffron extract (6.5 mg/kg, *per os*) did not reduce LPS-induced sickness behavior, preserving therefore this adaptive behavioral response essential for host defense. However, it interfered with delayed changes of expression of cytokines, chemokines and markers of microglial activation measured 24 h post-LPS treatment in key brain areas for behavior and mood control (frontal cortex, hippocampus, striatum). Importantly, this pretreatment also counteracted by that time the impact of LPS on several neurobiological processes contributing to inflammation-induced emotional alterations, in particular the activation of the kynurenine pathway, assessed through the expression of its main enzymes, as well as concomitant impairment of serotonergic and dopaminergic neurotransmission.

**Conclusion:**

Altogether, this study provides important clues on how saffron extract interferes with brain function in conditions of immune stimulation and supports the relevance of saffron-based nutritional interventions to improve the management of inflammation-related comorbidities.

## 1. Introduction

Over the past decades, a growing body of research has documented the dual nature of the impact of inflammation on brain function (1–4). When the innate immune system is challenged, inflammatory cytokines that are produced within the brain by activated microglia help the host fight infections, in particular by coordinating a large set of adaptive behavioral changes collectively referred to as sickness behavior (2, 4). However, sustained inflammation can also become deleterious and participate instead to the pathophysiology of many medical conditions, including the development of neuropsychiatric symptoms (1, 3, 5, 6). Therefore, identifying relevant approaches to selectively counter the harmful effects of inflammation is a major public health challenge.

Inflammation-driven neuropsychiatric alterations have been shown to occur when the activity of specific brain metabolic pathways, namely the kynurenine (KYN) and tetrahydrobiopterin (BH4) pathways, is changed and monoamine neurotransmission, which is crucial for the control of behavior and mood (7, 8), is impaired (1, 3, 5). Activation of indoleamine 2,3-dioxygenase (IDO), the first and limiting enzyme of the KYN pathway, leads to the synthesis of KYN from tryptophan at the expense of serotonin (5-HT) of which it is the precursor. In addition, downstream enzymes of the pathway that are also activated by cytokines further metabolize KYN into different toxic derivatives, which promote in turn oxidative stress and glutamate-related neurotoxicity (3, 5). Recent clinical reports have linked the generation of those neurotoxic metabolites to the severity of inflammation-related depressive symptoms (9, 10). Supporting these findings, preclinical studies have shown that activation of the KYN pathway by an inflammatory inducer, such as the lipopolysaccharide (LPS), plays a causal role in the induction of depressive-like behavior (11–13). Concurrently, cytokines also dysregulate the BH4 pathway (14–16), BH4 being an essential cofactor for several enzymes responsible for monoamine synthesis, particularly dopamine (DA) whose reduced levels are associated with depressive symptoms related to fatigue, anhedonia or decreased motivation (7). By increasing the activity of the first and limiting enzyme of this pathway, the GTP-cyclohydroxylase-1 (GTPCH1), cytokines favor the production of toxic derivatives, at the cost of BH4, hence decreasing its bioavailability for monoamine production (5, 15). Supporting the link between dysregulated BH4 pathway, impaired DA neurotransmission and behavioral alterations in inflammatory conditions, BH4 supplementation was recently shown to reduce alterations of DA-related behaviors in mice challenged with LPS (16). Altogether, these findings suggest that any strategy likely to interfere, beyond inflammation, with the activation of the KYN and BH4 pathways could hold great promise in helping to reduce neuropsychiatric symptoms related to inflammation.

In that context, nutritional interventions using natural dietary supplements with potential immunomodulatory and/or neuromodulatory properties could be useful (17–19) and mounting evidence particularly points to saffron as a promising candidate (20–26). This spice derived from stigmas of the flower *Crocus Sativus* L. is composed of different biologically active compounds contributing to its taste, but more importantly to its wide therapeutic effects, which interestingly include beneficial impact on mood. Experimentally, saffron or its active compounds have been shown to reduce depressive-like behavior in rodents (27–35). Similarly, administration of saffron extracts, either alone or in combination with standard antidepressants, improves depressive symptomatology in patients suffering from mild to moderate or severe symptoms of depression (36–42). A wide literature also sheds light on the immunomodulatory and anti-inflammatory properties of saffron in cell cultures or models of immune diseases (21, 23–26, 43, 44), as well as its ability to positively target neurobiological processes whose impairment may contribute to inflammation-related neuropsychiatric alterations, including monoamine neurotransmission, oxidative stress or neurogenesis (21, 30, 31, 33, 45–52). Noteworthy, we recently demonstrated that saffron supplementation modulates the expression of key enzymes of the KYN and BH4 pathways, whether under basal or stressful conditions (31). Based on these findings, we hypothesized that saffron extract administration may interfere with inflammation-induced neurobiological alterations and therefore be useful to reduce associated neuropsychiatric alterations.

The present study aims to test this hypothesis by investigating whether an oral administration of a standardized saffron extract previously shown to reduce depressive-like behavior (30, 31) was able to reverse the neurobiological alterations elicited by a systemic immune stimulation with LPS. This inflammatory inducer was chosen because it has been used for decades to study brain actions of cytokines and subsequently to unravel the mechanisms linking inflammation to depression (11–13, 53). We therefore measured first the impact of saffron extract pretreatment on LPS-induced sickness behavior, taken as a reflect of the induction of cytokines occurring over the first hours following the immune challenge. Second, we assessed its effect on the neurobiological processes known to underlie inflammation-related depressive-like behavior. We confirmed that saffron extract pretreatment interfered with these processes, which suggests that it may be useful to reduce the deleterious consequences of inflammation, while preserving the sickness behavior necessary to the host defense.

## 2. Materials and methods

### 2.1. Animals and treatments

On arrival, 8-week-old male C57BL/6 J mice (Janvier laboratories; Le Genest-Saint-Isle, France) were randomly divided into four groups (*n* = 14/group) matched for body weight. They were housed individually in an enriched (cardboard rodent homes and cotton nestlets) and controlled environment (22°C +/− 2°C, 40% humidity), and maintained under a normal 12/12 light/dark cycle (light on at 7 am) with free access to water and standard rodent chow (A04, SAFE, Augy, France). All procedures were conducted in accordance with the European legislation (260/63/EU) and were approved by the local and national Ethical Committees (APAFIS# 16873/16492).

As previously described (30), a saffron extract (SE) standardized in crocins, picrocrocins, safranal and kaempferol according to patent number #EP3490575 (Activ’Inside; Beychac-et-Caillau, France) and its vehicle (tap water) were administered orally using a flexible tube that was gently inserted into the digestive tract (mouse-adapted feeding probes 1.33x30mm, ECIMED; Boissy-Saint-Léger, France). The dose (6.25 mg/kg *per os*) and volume (10 mL/kg) of saffron extract used were selected based on previously published data (30, 54). Mice were handled and habituated to the administration procedure for several day before treatment in order to minimize stress reaction.

The solution of phenol-extracted lipopolysaccharide (LPS) from *Escherichia Coli* (serotype 0127:B8, Sigma; St. Louis, MO, US) was freshly prepared the day of test with endotoxin-free isotonic saline and intraperitoneally (ip) injected to half of the mice, the others receiving saline. The dose used (830 μg/kg) was selected based on its ability to induce a reliable activation of inflammatory processes and related neurobiological and behavioral changes (12, 53).

### 2.2. Experimental design

**Figure 1:**
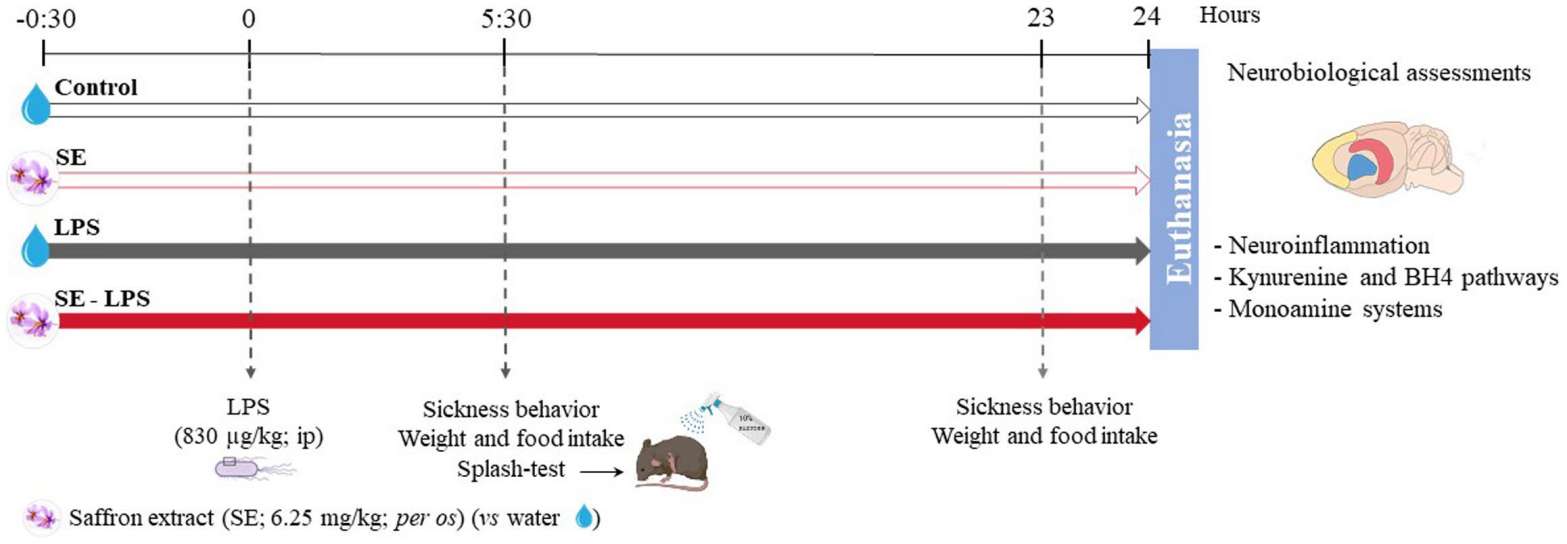
Experimental timeline and design. The impact of acute oral administration of saffron extract (6.25 mg/kg) was measured on behavioral (sickness behavior, splash-test), neuroinflammatory (brain expression of inflammatory factors) and neurobiological (KYN and BH4 pathways, monoamine systems) alterations induced by a lipopolysaccharide challenge (LPS, 830 μg/kg, ip). *n* = 14 mice/group.

The procedure included 4 different experimental groups (Figure 1). On the test day, mice received first an oral administration of SE or water, followed 30 min later by an ip injection of LPS or saline. This experimental design was chosen based on our previous results reporting a beneficial effect of similar SE pretreatment against stress-induced neurobiological and behavioral alterations (31). Body weight, food intake, and sickness behavior were assessed 5 h30 and 23 h after the LPS injection. Twenty-four hours post-LPS administration, mice were anesthetized with an ip injection of pentobarbital/lidocaine solution (300 and 30 mg/kg respectively). Once asleep, they were perfused with chilled PBS 1X for 2 min to remove all traces of blood from the tissues. Brains were then extracted from the skulls and frontal cortex (FCx), striatum (STR) and hippocampus (HPC) were carefully dissected from each half brain, immediately frozen with dry ice and stored at −80°C.

### 2.3. Sickness behavior assessment

Inflammation-induced sickness behavior includes several non-specific symptoms aiming to help the organism fighting back the infection. In LPS-treated mice, these symptoms usually developed within a few hours following the treatment and then progressively disappear. Sickness behavior was evaluated by calculating a sickness score that depends on the intensity (from 1 to 4) of three main symptoms: piloerection (normal/irregular/erected coat), ptosis (normal/half-closed/tearful/closed eyes) and general locomotion (normal/slow/huddled-up/lethargy) (55). Mice were scored during the peak of inflammation (5 h30) and 23 h post-treatment (Figure 1) in order to evaluate how sick they were and how well they recovered, respectively. A delta sickness score, whose amplitude reflects the degree of recovery, was also calculated as the difference between the scores at 23 h and 5 h30 post-treatment.

One of the symptoms classically found during sickness behavior being loss of self-care, we evaluated this parameter in more details using the splash-test. This test was performed in a soundproof room, essentially as previously described (56). A viscous 10% sucrose solution known to trigger grooming behavior was squirted on the dorsal coat of each mouse in their home cages. Latency to initiate grooming and its duration over the 5-min test were manually scored as an index of self-care. All behavioral assessments were performed by a trained observer blind to treatments.

### 2.4. Gene expression analysis

RT-qPCR was performed as previously described (30). Briefly, total RNAs were extracted from the collected structures using Trizol reagent (Invitrogen, Life Technologies, Villebon-sur-Yvette, France) and reverse transcribed into cDNAs by using the Superscript III reverse transcriptase (Invitrogen, Waltham, MA, US). The Taqman LightCycler® 480 Probes Master mix (Roche Diagnostics, Meylan, France) and its associated FAM-labeled Taqman Primers (ThermoFisher Scientific, Waltham, MA, US) were used to amplify the genes of interest from 2 μL of cDNAs at 20 μg/μL. All experiments were performed in duplicates. Fluorescence was measured using a LightCycler® 480 II system (Roche Diagnostics, Meylan, France). Data were analyzed with the comparative threshold cycle method and results were normalized with GAPDH as a house-keeping gene. Primer references are given in Supplementary Table S1.

### 2.5. Brain monoamines assessment

Brain 5-HT, DA and their metabolites, the 5-hydroxyindoleacetic acid (5-HIAA), dihydroxyphenylacetic acid (DOPAC) and homovanillic acid (HVA), were measured by HPLC-EC, essentially as previously described (30). Half structures of interest were lysed in 600 μL of fresh extraction buffer (4 × 1 min at 30 Hz) using a Tissue Lyser (Qiagen, Courtaboeuf, France). After homogenate centrifugation (16,000 g, 4°C, 20 min), the supernatants were centrifuged again into filtering tubes (1,600 g, 4°C, 2 min) and the final supernatants containing the monoamines stored at −80°C until use. For HPLC assay, 20 μL of the supernatant were injected into a chromatograph equipped with a 5 μm C18, 3×100 mm silica column (ACE, AIT France, Cormeilles-en-Parisis, France) and coupled to an electrochemical detection system (Antec Decade 2, CJ Lab, La Frette, France). Monoamines and their metabolites were identified through their retention times and quantified using the Chromeleon integration 6.8 software (Dionex, Sunnyvale, CA, US). Results were expressed in nmoles/g of tissue.

### 2.6. Statistical analysis

Data were analyzed using the software Statistica 6 (StatSoft, Tulsa, OK, US) and the Graphpad Outlier Calculator (Prism, San Diego CA, US) to identify statistical outliers, which were excluded from the analyses. Two-way ANOVAs, followed by a LSD Fisher post-hoc when appropriate, were performed whenever the normality, independence and homogeneity of variances conditions were fulfilled. Otherwise, data were analyzed using Kruskal-Wallis ANOVA and multiple comparison of the mean ranks if significant. Potential changes over time were analyzed by a repeated measures ANOVA for body weight and non-parametric tests for food intake and sickness score. Student t-test or Mann–Whitney *U*-test were performed to evaluate the significance between two groups. Graphs were presented as mean ± SEM and *p* values ≤0.05 denote statistical significance.

A Principal Components Analysis (PCA) was applied using R version 3.3.0 (FactoMineR package) in order to separate mice of each group according to their respective inflammatory and neurobiological profile. The principal components (PC) generated represent linear combinations of the initial variables. Factor loadings obtained for each variable reflect its correlation with the PC, variables with the highest loading values (≥0.25) contributing the most to the PC construct.

## 3. Results

### 3.1. Impact of saffron extract on LPS-induced sickness behavior

**Figure 2:**
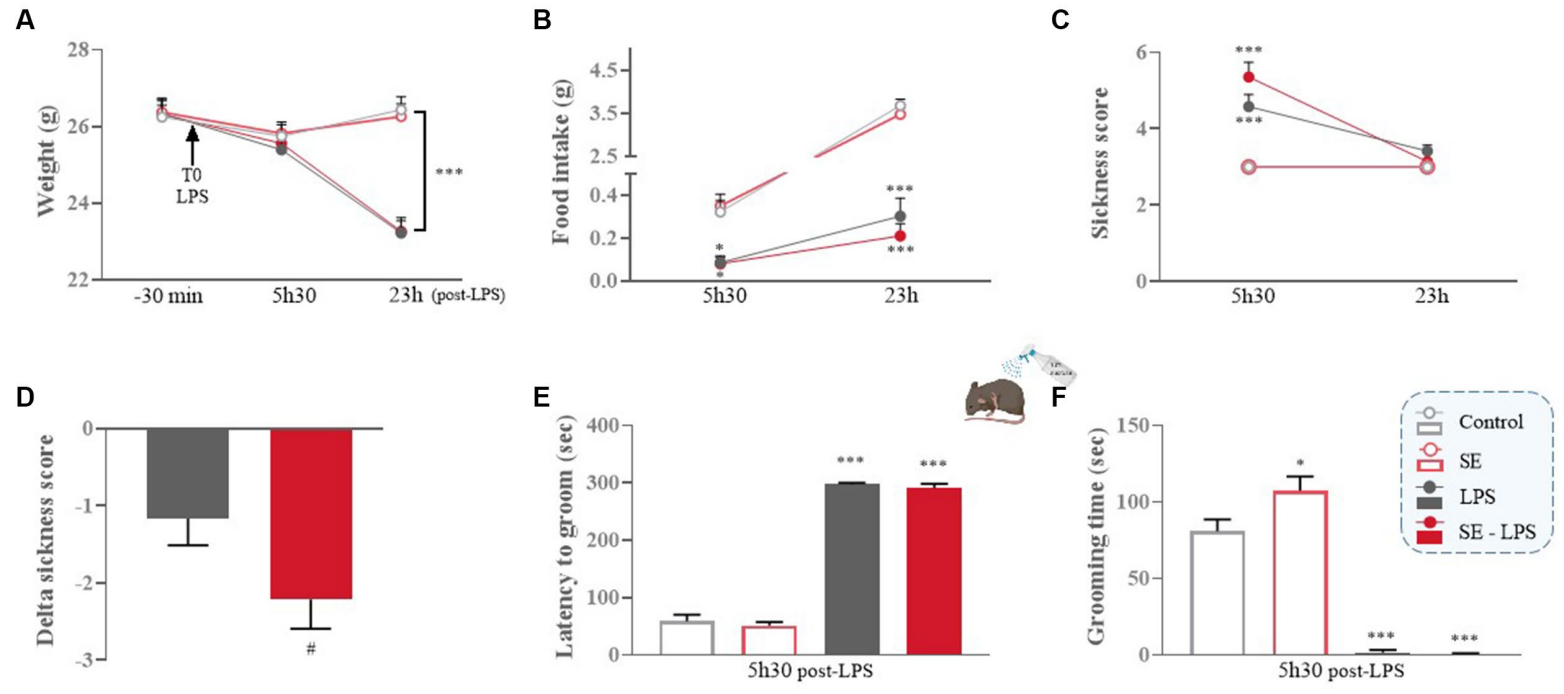
Effects of oral saffron extract administration (6.25 mg/kg) on LPS-induced sickness behavior (830 μg/kg, ip). **(A)** Body weight (g) measured 30 min before LPS injection (T0) and 5 h30 and 23 h after; **(B)** Food intake (g) measured 5 h30 and 23 h after LPS injection; **(C)** Total sickness score assessed 5 h30 and 23 h after LPS injection; **(D)** Delta sickness scores (between the two timepoints) in LPS-treated groups; **(E)** Latency to groom (sec) and **(F)** Duration of grooming (sec) in the splash-test conducted 5 h30 after LPS injection. Results are shown as mean ± SEM. ^*^ *p* ≤ 0.05, ^***^ *p* ≤ 0.001 vs. Control; ^#^ *p* ≤ 0.05 vs. LPS.

We first checked whether saffron extract (SE) reduced LPS-induced sickness behavior and related changes of body weight and food intake. As expected, body weight was progressively reduced in LPS-treated mice as compared to their non-treated counterparts [LPS: *F*(1,49) = 11.53; *p* ≤ 0.001; Time x LPS: *F*(2,98) = 39; *p* ≤ 0.001; Figure 2A], the difference reaching significance at 23 h post-LPS (LPS and SE-LPS vs. Control: *p* ≤ 0.001), consistent with the reduction in food intake shown by LPS-treated mice compared to the control group 5 h30 (LPS vs. Control: *p* ≤ 0.05 and SE-LPS vs. Control: *p* ≤ 0.01; Figure 2B) and 23 h after treatment (LPS and SE-LPS vs. Control: *p* ≤ 0.001). Pretreatment with SE had no impact on body weight or food intake regardless of LPS challenge.

As anticipated, control mice and mice only treated with SE did not develop sickness behavior, as confirmed by their low sickness score (Figure 2C). On the contrary, this score was significantly increased in LPS and SE-LPS-treated mice 5 h30 after treatment (LPS and SE-LPS vs. Control: *p* ≤ 0.001), indicating the induction of a strong sickness behavior which was not prevented by saffron. Then, all LPS-treated mice progressively recovered, as confirmed by the lack of significant differences in the multiple groups comparison analysis of the sickness scores measured 23 h post-LPS. Of note, although sickness scores were not significantly different between LPS-and SE-LPS-treated mice at the two time points, the delta sickness score was bigger in the SE-LPS group (*p* = 0.05; Figure 2D), suggesting that saffron may potentially facilitate recovery.

To further investigate sickness symptoms, particularly reduced self-care, changes in latency and duration of grooming were assessed in the splash-test at the peak of the sickness phase (5 h30 post-LPS). The LPS challenge drastically increased the latency to groom [*H*(3,54) = 43.12; *p* ≤ 0.001; LPS vs. Control: *p* ≤ 0.001; Figure 2E] despite SE pretreatment (SE-LPS vs. Control: *p* ≤ 0.001). All LPS-treated mice also spent less time grooming than controls [*H*(3,54) = 44.54; *p* ≤ 0.001; LPS and SE-LPS vs. Control: *p* ≤ 0.001; Figure 2F]. Besides, the 2-by-2 groups comparison shows that saffron increased the duration of grooming when administered alone (SE vs. Control: *p* ≤ 0.05), suggesting it may beneficially impact mice welfare under basal conditions.

### 3.2. Impact of saffron extract on LPS-induced neuroinflammation

The impact of SE on LPS-induced activation of brain inflammatory processes was then assessed by measuring the expression of genes coding for different cytokines, chemokines and markers of microglial activation in the FCx, STR and HPC. These brain areas were chosen because they are both sites for induction of inflammation and control of related behavioral alterations (53, 57, 58). Moreover, we recently reported neurobiological changes in these structures after SE administration (30, 31).

**Figure 3:**
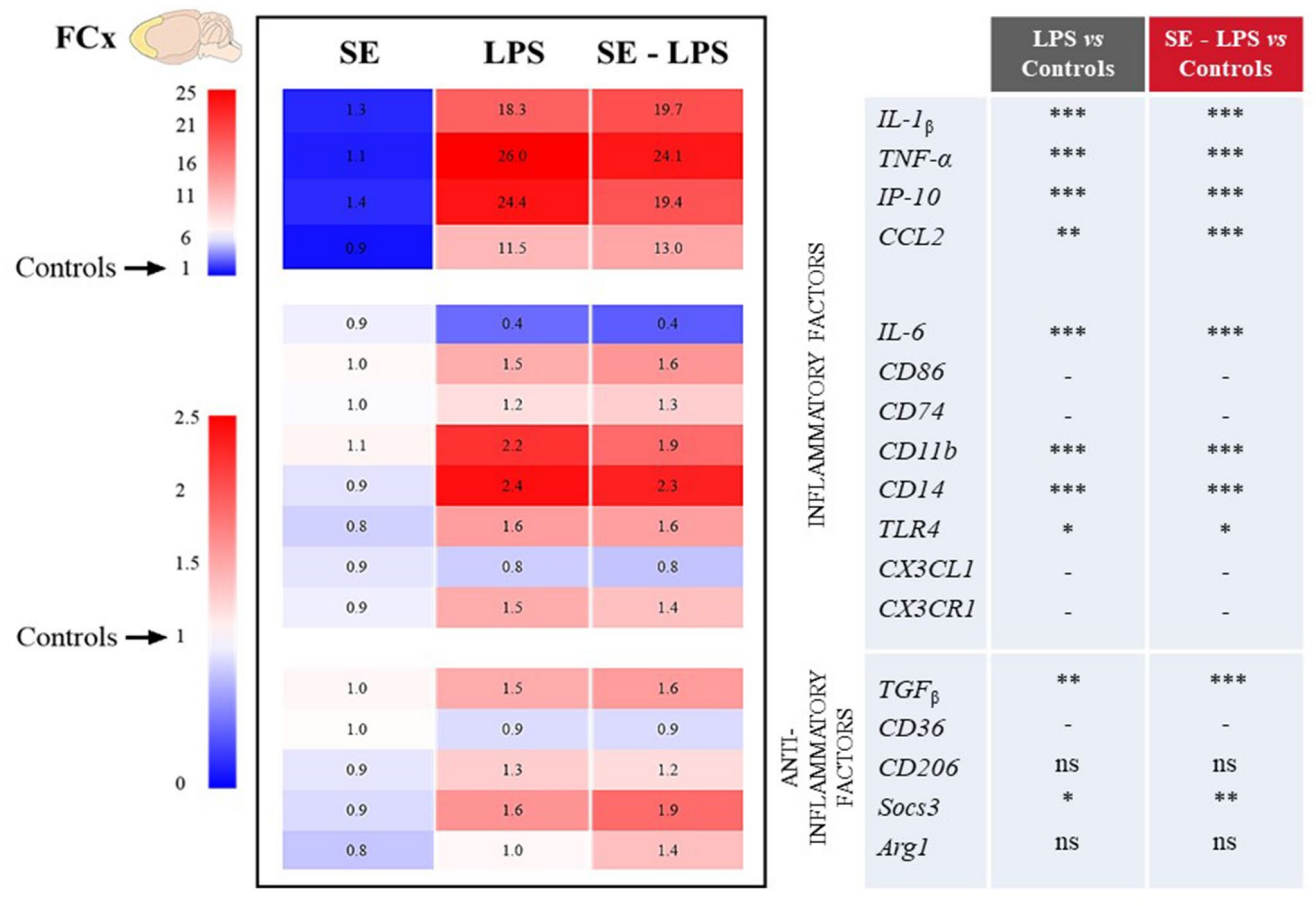
Effects of oral saffron extract administration (6.25 mg/kg) on LPS-induced neuroinflammation (830 μg/kg, ip) in the FCx. Heatmaps displaying relative gene expression (as compared to Controls = 1) of inflammatory and anti-inflammatory factors measured 24 h after the LPS challenge. Numbers on the heatmaps represent the mean foldchange of each group. In the table, the columns represent the results of multiple comparisons following significant Kruskal-Wallis test. ^*^ *p* ≤ 0.05, ^**^ *p* ≤ 0.01, ^***^ *p* ≤ 0.001, ns = not significant, − = not determined (parametric data with no significant SE x LPS interaction). *IL-1_β_*: Interleukin-1_β_; *TNF-α*: Tumor Necrosis Factor-α; *IP-10*: Interferon gamma-induced Protein 10; *CCL2*: CC motif Chemokine Ligand 2; *IL-6*: Interleukin-6; *CD86/74/11b/14/36/206*: Clusters of Differentiation; *TLR4*: Toll-Like Receptor 4; *CX3CL1*: CX3C motif Chemokine Ligand 1; *CX3CR1*: CX3C motif Chemokine Receptor 1; *TGF_β_*: Transforming growth factor-β; *Socs3*: Suppressor of cytokine signaling 3; *Arg1*: Arginase 1.

**Figure 4:**
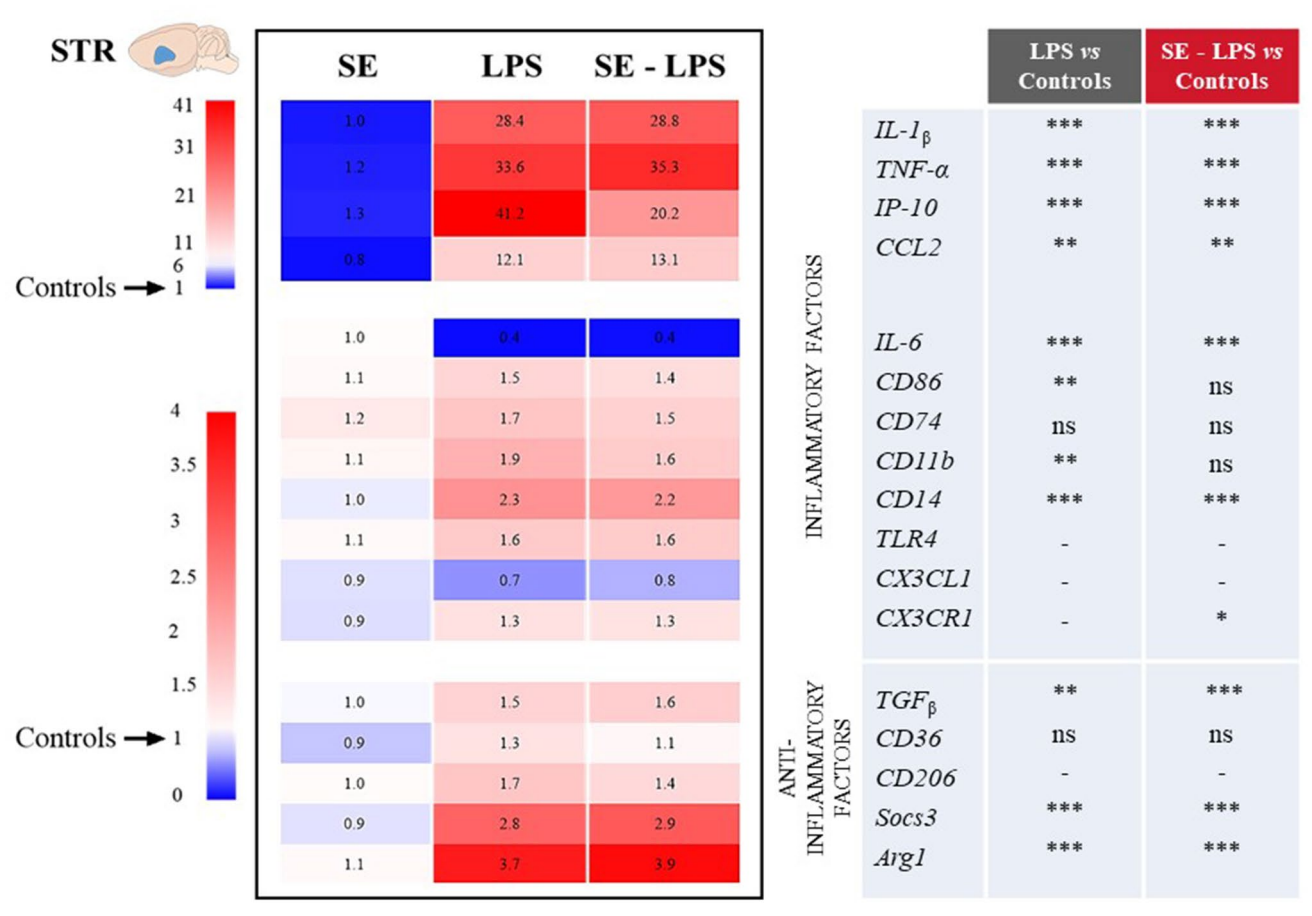
Effects of oral saffron extract administration (6.25 mg/kg) on LPS-induced neuroinflammation (830 μg/kg, ip) in the STR. See legend of Figure 3 for details. ^*^ *p* ≤ 0.05, ^**^ *p* ≤ 0.01, ^***^ *p* ≤ 0.001, ns = not significant, − = not determined (parametric data with no significant SE x LPS interaction).

**Figure 5:**
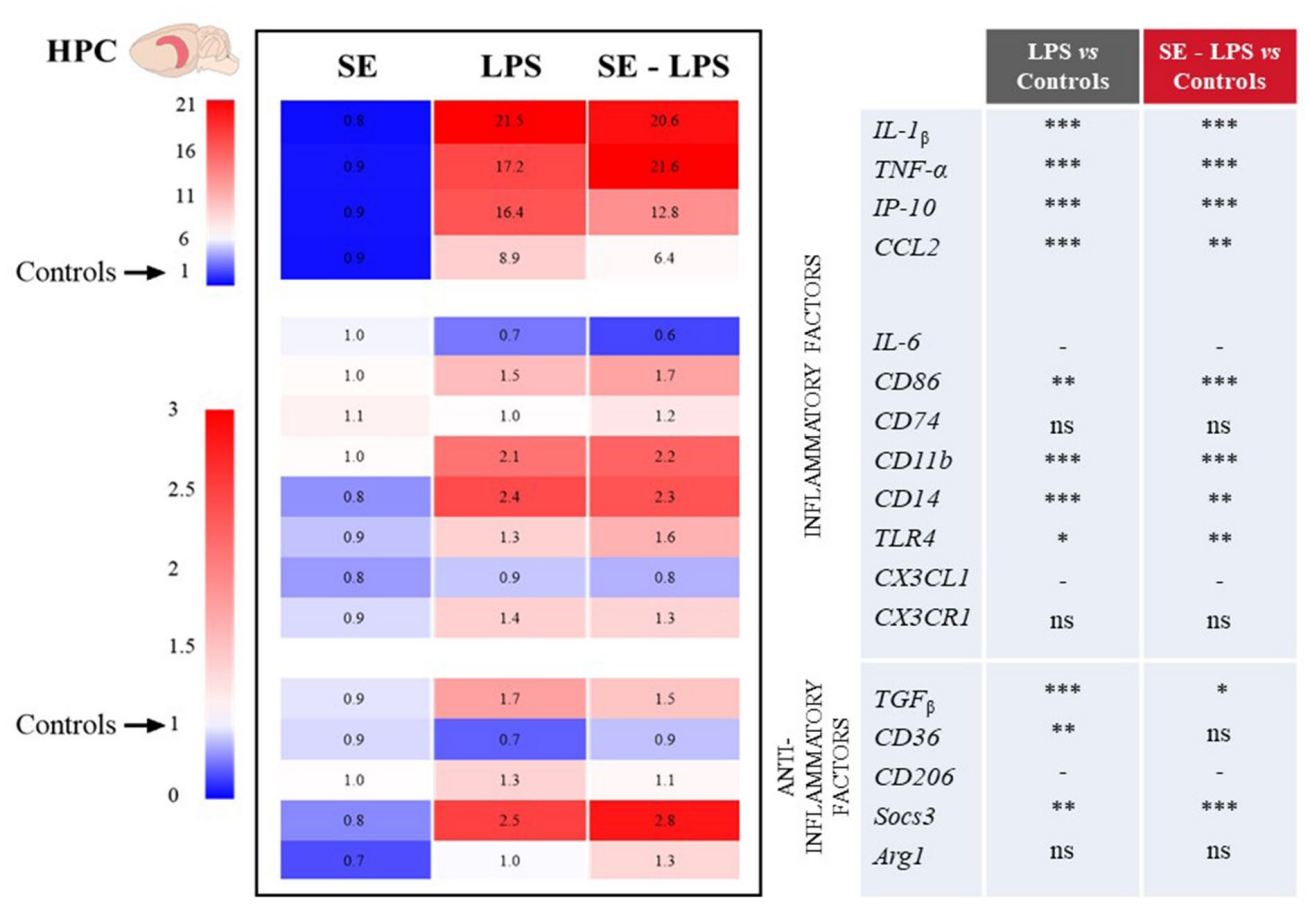
Effects of oral saffron extract administration (6.25 mg/kg) on LPS-induced neuroinflammation (830 μg/kg, ip) in the HPC. See legend of Figure 3 for details. ^*^ *p* ≤ 0.05, ^**^ *p* ≤ 0.01, ^***^ *p* ≤ 0.001, ns = not significant, − = not determined (parametric data with no significant SE x LPS interaction).

Consistent with the development of sickness behavior, LPS-induced neuroinflammation was detected in the three brain areas 24 h after treatment, although with some specificities. Compared to controls, LPS-treated mice exhibited significantly higher relative expression of interleukin-1β (IL-1β), tumor necrosis factor-α (TNF-α), interferon gamma-induced protein 10 (IP-10) and CC motif chemokine ligand 2 (CCL2) in all brain areas. This was true whether mice were pretreated with SE or not (see multiple comparisons in Figures 3–5), excepted for IP-10 overexpression in the STR that was attenuated by this pretreatment (SE-LPS vs. LPS: *p* ≤ 0.05; Figure 4). IL-6 expression was reduced in the FCx [*H*(3,50) = 37.04; *p* ≤ 0.001; Figure 3], STR [*H*(3,49) = 33.73; *p* ≤ 0.001; Figure 4] and HPC [*F*(1,47) = 7.72; *p* ≤ 0.01; Figure 5] of LPS-and SE-LPS-treated mice. Regarding markers of microglial activation, LPS upregulated the expression of cluster of differentiation-86 (CD86) [*F*(1,48) = 44.34; *p* ≤ 0.001], CD74 [*F*(1,46) = 8.27; *p* ≤ 0.01] and CD11b [*H*(3,51) = 37.71; *p* ≤ 0.001] in the FCx (Figure 3), as well as CD86 [*H*(3,52) = 28.42; *p* ≤ 0.001] and CD11b [*H*(3,53) = 38.33; *p* ≤ 0.001] in the HPC (Figure 5), regardless of SE pretreatment. Expression of CD86 [*H*(3,49) = 13.39; *p* ≤ 0.01] and CD11b [*H*(3,49) = 15.19; *p* ≤ 0.001] was also increased by LPS in the STR, but this was prevented by saffron (SE-LPS vs. Control: *p* > 0.1; Figure 4). The 2-by-2 comparison shows that LPS also enhanced striatal CD74 expression, but only in the group without saffron pretreatment (LPS vs. Control: *p* ≤ 0.01; Figure 4). All LPS-treated mice also exhibited, in the 3 brain areas, increased expression of the co-receptors involved in LPS detection, CD14 [FCx: *H*(3,52) = 37.95; STR: *H*(3,48) = 35.23; HPC: *H*(3,50) = 38.30; *p* ≤ 0.001] and toll-like receptor-4 (TLR4) [FCx: *H*(3,48) = 26.03; HPC: *H*(3,50) = 27.47; *p* ≤ 0.001; STR: *F*(1,42) = 80.82; *p* ≤ 0.001]. Of note however, hippocampal CD14 expression was globally downregulated as compared to controls in SE-treated mice (SE vs. Control: *p* ≤ 0.01; Figure 5). Lastly, expression of CX3C motif chemokine ligand 1 (CX3CL1) and receptor 1 (CX3CR1) was upregulated by LPS in the FCx [*F*(1,45) = 8.57; *p* ≤ 0.01 and *F*(1,44) = 28.83; *p* ≤ 0.001 respectively; Figure 3] and STR [*F*(1,44) = 11.36; *p* ≤ 0.001 and *H*(3,46) = 13.64; *p* ≤ 0.01 respectively; Figure 4].

Concurrently, we measured the expression of several important anti-inflammatory factors, such as transforming growth factor (TGFβ), CD36, CD206, suppressor of cytokine signaling (Socs3) and arginase type-1 (Arg1). LPS increased expression of TGFβ [FCx: *H*(3,49) = 31.21; STR: *H*(3,47) = 26.66; HPC: *H*(3,48) = 24.03; *p* ≤ 0.001] and Socs3 [FCx: *H*(3,51) = 26.19; STR: *H*(3,46) = 32.88; HPC: *H*(3,51) = 38.94; *p* ≤ 0.001], independently of SE pretreatment (Figures 3–5). CD206 expression was significantly upregulated by LPS in the STR [*F*(1,45) = 3.45; *p* ≤ 0.0001; Figure 4] and tended to be increased in the HPC [*F*(1,46) = 3.06; *p* = 0.08; Figure 5], what was confirmed by the 2-by-2 comparison analysis (LPS vs. Control: *p* ≤ 0.05). Still in the HPC, LPS decreased CD36 expression [*H*(3,47) = 11.43; *p* ≤ 0.01; Figure 5] unless mice were pretreated with SE (SE-LPS vs. Control: *p* > 0.1). Lastly, expression of Arg1 was increased by LPS only in the STR [*H*(3,44) = 28.47; *p* ≤ 0.001; Figure 4]. However, the group comparisons indicate that SE pretreatment tended to reduce Arg1 expression in the FCx and HPC in the absence of LPS treatment (SE vs. Control: *p* = 0.065 and *p* = 0.054 respectively; Figures 3, 5), while it increased its expression in the FCx of LPS-treated mice (SE-LPS vs. Control and vs. LPS: *p* ≤ 0.05; Figure 3). Altogether, these results showed that SE pretreatment interfered with LPS-induced activation of neuroinflammatory processes 24 h after treatment by targeting different inflammatory and/or anti-inflammatory factors depending on the brain area.

### 3.3. Impact of saffron extract on LPS-induced activation of KYN and BH4 pathways

**Figure 6:**
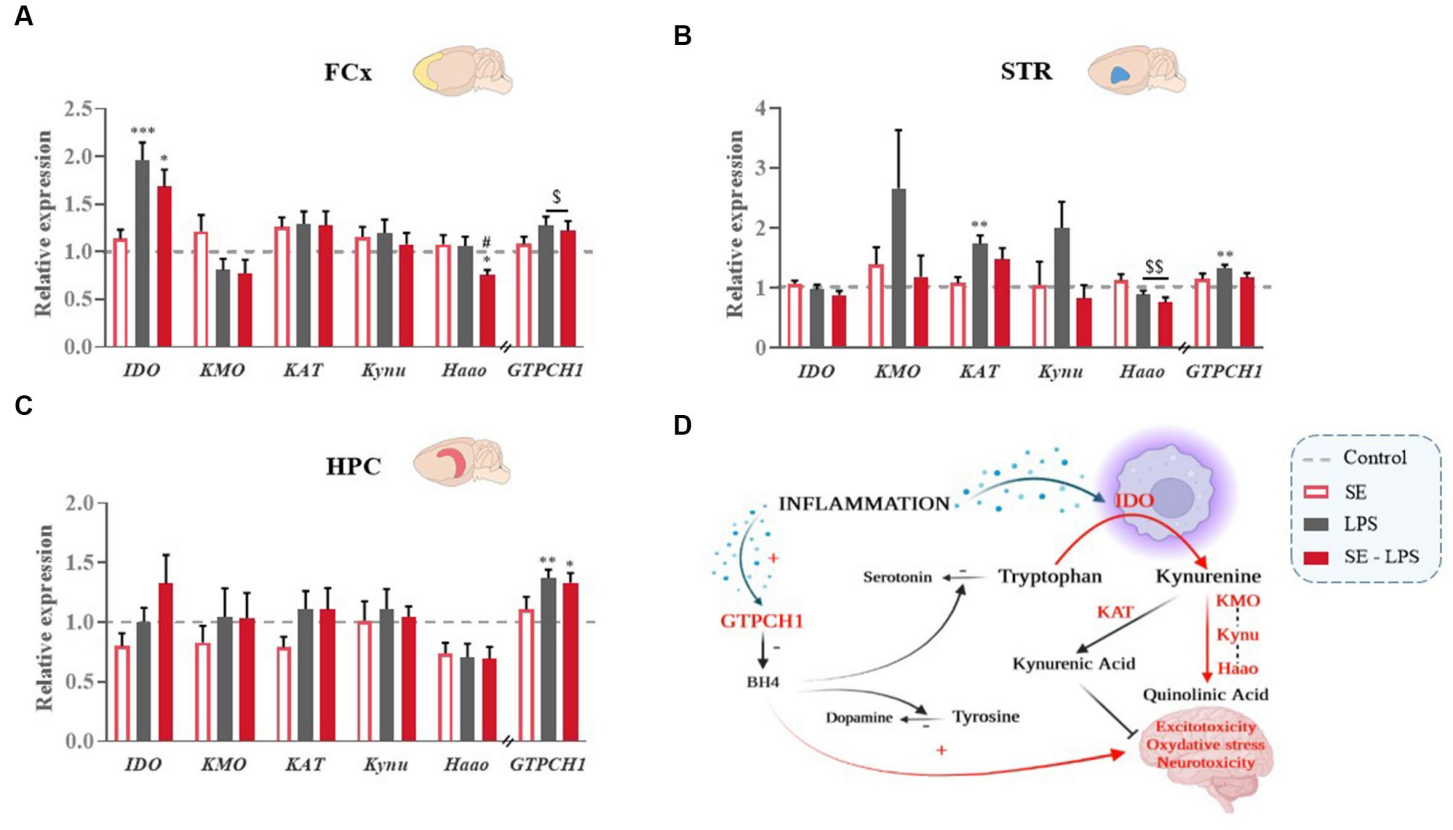
Effects of oral saffron extract administration (6.25 mg/kg) on LPS-induced activation of KYN and BH4 pathways (830 μg/kg, ip). Relative expression of Indoleamine 2,3-dioxygenase (*IDO*), Kynurenine 3-Monooxygenase (*KMO*), Kynurenine Aminotransferase (*KAT*), Kynureninase (*Kynu*), 3-Hydroxyanthranilate 3,4-Dioxygenase (*Haao*) and GTP-Cyclohydrolase 1 (*GTPCH1*) measured in the **(A)** FCx, **(B)** STR, and **(C)** HPC 24 h after the LPS challenge. **(D)** Schematic representation of the activation of the KYN and BH4 pathways in inflammatory conditions. Data are represented as foldchanges normalized to the control group (baseline = 1). Results are shown as mean ± SEM. ^*^ *p* ≤ 0.05, ^**^ *p* ≤ 0.01, ^***^ *p* ≤ 0.001 vs. Control; ^#^ *p* ≤ 0.05 vs. LPS; ^$^ *p* ≤ 0.05, ^$$^ *p* ≤ 0.01 global LPS effect.

LPS-induced brain inflammation is well-known to change the activity of the KYN and BH4 pathways (Figure 6D), which in turn contribute to inflammation-related neurotoxicity and monoamine alterations (1, 5). The potential impact of SE pretreatment on these processes was therefore assessed by measuring the expression of key enzymes of each pathway.

No significant pretreatment or treatment effect was detected regarding the expression of KYN enzymes in the HPC (Figure 6C). In the FCx, IDO expression was significantly increased by LPS [*H*(3,52) = 20.45; *p* ≤ 0.001; Figure 6A], although to a lesser extent when mice were pretreated with SE (SE-LPS vs. Control: *p* ≤ 0.05; LPS vs. Control: *p* ≤ 0.001). Similarly, expression of 3-hydroxyanthranilate 3,4-dioxygenase (Haao), which significantly differed between the groups [*H*(3,52) = 8.9; *p* ≤ 0.05], was reduced in SE-LPS-treated mice as compared to controls and LPS-treated mice (*p* ≤ 0.05). In the STR, LPS decreased Haao expression [*F*(1,44) = 8.01; *p* ≤ 0.01; Figure 6B] regardless of SE pretreatment and increased kynurenine aminotransferase (KAT) expression, but only in non-pretreated mice [H(3,48) = 15.02; *p* ≤ 0.001; LPS vs. Control: *p* ≤ 0.01; SE-LPS vs. Control: *p* > 0.1; Figure 6B]. Likewise, kynurenine 3-monooxygenase (KMO) and kynureninase (Kynu) expression appeared to be upregulated by LPS when administrated alone, but this did not reach statistical significance (Figure 6B).

Concerning the BH4 pathway, the expression of its first enzyme, GTPCH1, was enhanced by LPS in the FCx [*F*(1,47) = 6.53; *p* ≤ 0.05; Figure 6A], STR [*F*(1,43) = 6.17; *p* ≤ 0.05; Figure 6B] and HPC [*H*(3,53) = 15.3; *p* ≤ 0.05; LPS vs. Control: *p* ≤ 0 0.01; SE-LPS vs. Control: *p* ≤ 0.05; Figure 6C]. SE pretreatment only prevented this induction in the STR [LPS x SE: *F*(1,43) = 4.85; *p* ≤ 0.05; LPS vs. Control: *p* ≤ 0.01; SE-LPS vs. Control: *p* > 0.1; Figure 6B]. Taken together, these results highlight the ability of SE pretreatment to modulate the impact of LPS on the KYN and BH4 pathways.

### 3.4. Impact of saffron extract on LPS-induced modulation of monoamine neurotransmission

**Figure 7:**
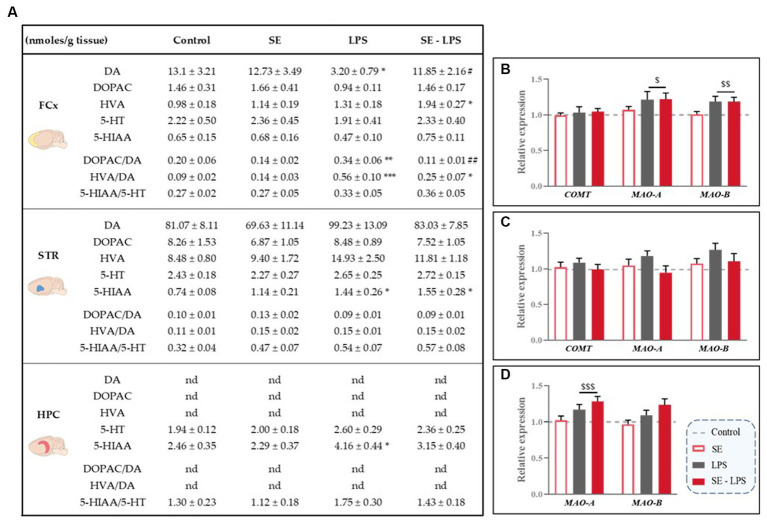
Effects of oral saffron extract administration (6.25 mg/kg) and LPS-induced changes of brain concentrations of monoamines and their metabolites (830 μg/kg, ip). **(A)** Levels of monoamines (DA, 5-HT) and their metabolites (DOPAC, HVA and 5-HIAA; nmoles/g of tissue) measured in the FCx, STR and HPC 24 h after the LPS challenge. Relative gene expression plotted as foldchanges normalized to the control group (baseline = 1) of Catechol-O-methyltransferase (*COMT*), Monoamine Oxidase-A (*MAO-A*) and *MAO-B* measured in the **(B)** FCx, **(C)** STR, and **(D)** HPC. Results are shown as mean ± SEM. nd: not detectable. ^*^ *p* ≤ 0.05, ^**^ *p* ≤ 0.01, ^***^ *p* ≤ 0.001 vs. Control; ^#^ *p* ≤ 0.05, ^##^ *p* ≤ 0.01 vs. LPS; ^$^ *p* ≤ 0.05, ^$$^ *p* ≤ 0.01, ^$$$^ *p* ≤ 0.001 global LPS effect.

Based on the aforementioned data and previous studies reporting positive modulation of monoamine neurotransmission by saffron in other experimental conditions (30, 31), we checked whether this pretreatment may improve LPS-induced monoamine impairments. Results are shown in Figure 7A.

Levels of DA and its metabolites were undetectable in the HPC and similar in all groups in the STR, as well as striatal expression of the DA degradation enzymes (Figure 7C). On the contrary, DA concentrations differed among the experimental groups in the FCx [*H*(3,50) = 8.54; *p* ≤ 0.05], with LPS-treated mice displaying lower DA levels than SE-LPS mice (*p* ≤ 0.05). DA levels were also significantly reduced in the LPS group as compared to controls (*p* ≤ 0.05), as revealed by the group-by-group comparison. Consistent with this, both cortical DOPAC/DA [*H*(3,44) = 13.8; *p* ≤ 0.01] and HVA/DA [*H*(3,45) = 18.35; *p* ≤ 0.001] ratios were significantly higher in LPS than control groups (LPS vs. Control: *p* ≤ 0.01 and *p* ≤ 0.001 respectively). Interestingly, SE pretreatment normalized DOPAC/DA ratio (SE-LPS vs. Control: *p* > 0.1) and tended to blunt LPS-induced increase in HVA/DA ratio, although it was still significantly different from controls (SE-LPS vs. Control: *p* ≤ 0.05). This is likely due to the slight increase in HVA concentrations displayed by SE-LPS mice [*H*(3,51) = 10.37; *p* ≤ 0.05; SE-LPS vs. Control: *p* ≤ 0.05]. Supporting further increased cortical DA turnover in response to LPS, it also significantly upregulated monoamine oxidase-A (MAO-A) and MAO-B expression [*F*(1,47) = 5.24; *p* ≤ 0.05 and *F*(1,47) = 10.23; *p* ≤ 0.01 respectively; Figure 7B].

Regarding 5-HT-related measures, no significant impact of LPS and/or SE administration was observed in the FCx, while in the STR LPS-treated mice displayed a significant increase in 5-HIAA levels whether or not they received SE pretreatment [*H*(3,47) = 11.76; *p* ≤ 0.01; LPS and SE-LPS vs. Control: *p* ≤ 0.05]. On the contrary, hippocampal 5-HIAA levels were selectively enhanced by LPS when administered alone [*H*(3,46) = 10.6; *p* ≤ 0.05; LPS vs. Control: *p* ≤ 0.05; SE-LPS vs. Control: *p* > 0.1], suggesting that SE pretreatment may interfere with LPS-induced 5-HT degradation, although it did not prevent LPS-induced overexpression of the 5-HT degradation enzyme MAO-A [*F*(1,47) = 13.12; *p* ≤ 0.001; Figure 7D]. In summary, these results show that beyond its impact on LPS-induced inflammation and enzymatic pathways activation, pretreatment with SE was also able to counteract the associated dysregulations of monoamine neurotransmission.

### 3.5. Global impact of saffron extract on LPS-induced brain alterations

**Figure 8:**
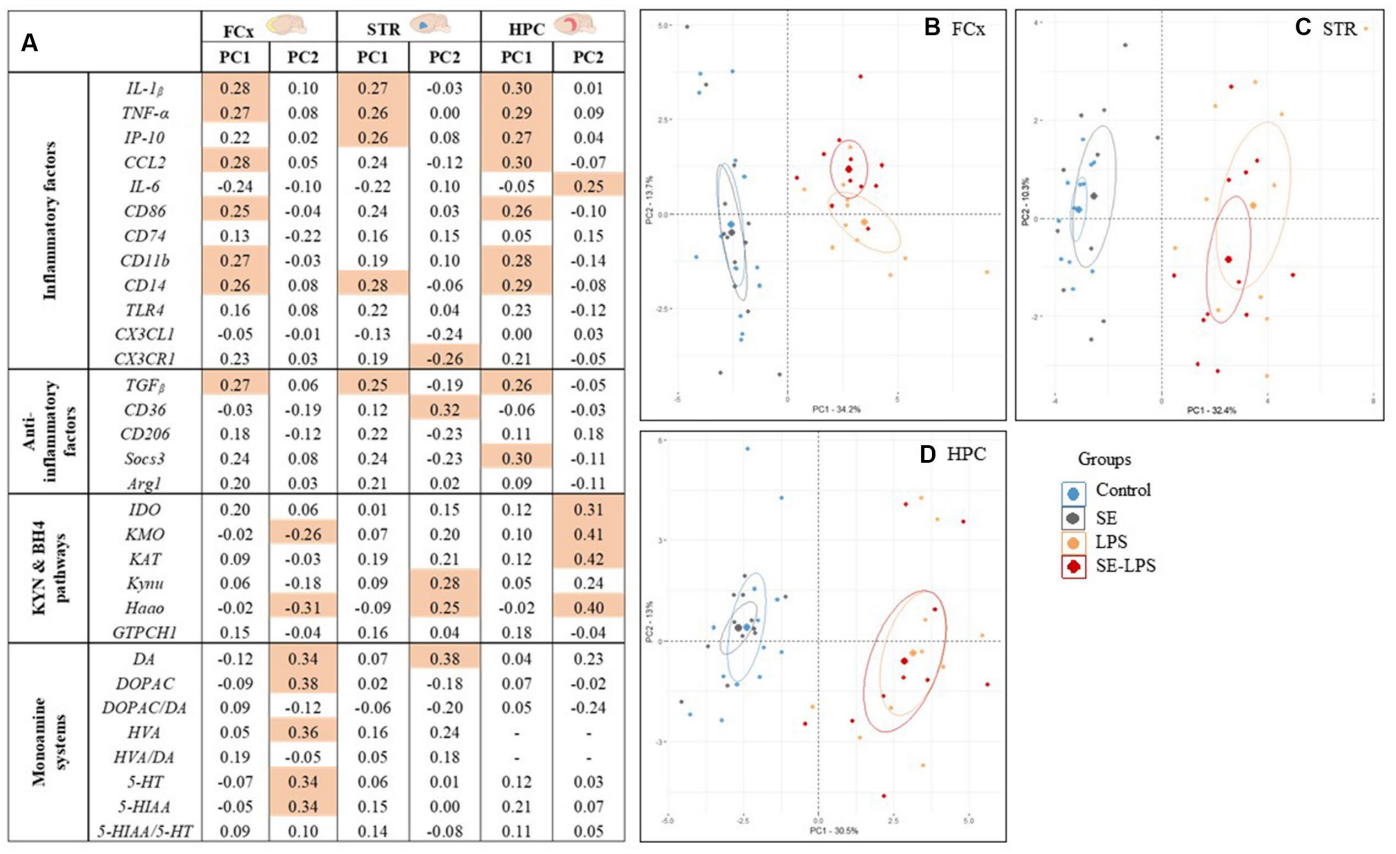
Principal Component Analysis (PCA) calculated for each brain area considering all the inflammatory and neurobiological parameters measured 24 h after the LPS challenge. **(A)** Factor loadings obtained for each variable and that reflect its correlation with the principal component (PC) score from the PCA. Shaded cells indicate the dominant variables, i.e., those with the highest loading values (≥ 0.25) within each PC. The loadings described how much each variable contributes to the PC; Individuals maps of PCA analysis for the **(B)** FCx, **(C)** STR, and **(D)** HPC.

Finally, a PCA was performed to visualize the global impact of SE pretreatment on the inflammatory and neurobiological profile displayed by the different experimental groups. In the FCx, STR and HPC, the 1st principal component (PC) explained more than 30% of the total variance (Figures 8B–D). Interestingly, PC1 was associated with higher levels of inflammatory factors (Figure 8A) and a highly significant LPS effect (*p* < 0.001) in the three brain areas, as well as a pretreatment by treatment interaction (*p* < 0.05) in the STR. As shown in the different individuals maps (Figures 8B–D), LPS-untreated and LPS-treated mice displayed negative and positive average scores for PC1, respectively, and they were therefore clearly dissociated. Importantly, while the distribution of individuals was always essentially comparable between the Control and SE groups, saffron pretreatment changed the profile of mice challenged with LPS, this effect depending however on the brain area. In the HPC, PC2 explained 13% of the total variance and revealed a highly positive score for KYN enzymes expression (Figure 8A). Nevertheless, there was a clear overlap between the scatter of individuals from the two LPS-treated groups (Figure 8D), in agreement with the lack of SE-induced modulation of KYN pathway activation reported in this brain area (Figure 6C). On the contrary, the PCA individuals score plots showed that PC2 separated LPS and SE-LPS groups in the FCx (Figure 8B) and to a lesser extent in the STR (Figure 8C). PC2 accounted for 13.7 and 10.3% of the total variance, respectively, and was related in both cases to KYN pathway and monoamine neurotransmission (restricted to DA in the STR; Figure 8A). Importantly, the average scores of SE-LPS groups for PC2 were always shifted to the side associated with lower KYN pathway activation and higher monoamine neurotransmission.

Altogether, these findings nicely illustrate the beneficial impact of saffron pretreatment on the different brain alterations occurring in response to a systemic immune challenge and notably involved in inflammation-driven neuropsychiatric alterations.

## 4. Discussion

Identifying new strategies to circumscribe the deleterious consequences of neuroinflammation on brain function is a major health concern due to its role in the pathophysiology of many medical conditions, including neuropsychiatric disorders. The present study provides valuable data relevant to this issue by showing that oral administration of a saffron extract modulates the neuroinflammatory response to an LPS challenge and its impact on several neurobiological processes known to contribute to LPS-induced emotional alterations. In addition, we highlighted the ability of saffron extract to act at different levels of the cascade of events leading from cytokine production to impairment of monoamine neurotransmission.

Chronic exposure to stressful environmental factors, inflammation inducers, or even their combination, is the primary paradigm used in many preclinical models of inflammation-driven depression (3, 35, 63–67). In the present study, LPS-treated mice exhibited the expected marked sickness behavior and anorexia, which are adaptive behavioral responses essential for the body to fight infection (1, 2, 4). SE pretreatment did not interfere with these responses, suggesting that it did not change the underlying early induction of brain inflammatory cytokines (mainly IL-1β, TNF-α and IL-6), which is consistent with what was found 24 h post-treatment. It could be argued that using higher doses of SE and/or changing the administration schedule could achieve an effect. However, a significant improvement of stress-induced neurobiological and behavioral alterations was previously reported by applying the current experimental procedure (31). Moreover, while SE pretreatment did not reduce the intensity of sickness behavior, it seemed to promote its resolution. Indeed, the overtime change in sickness score was larger in SE-LPS-treated mice, which also displayed blunted activation of inflammatory processes and related neurobiological impairments 24 h post-LPS treatment. Together, these findings strengthen the assumption of a preferential impact of saffron on late rather than early stages of the neuroinflammatory responses to LPS, which may be particularly valuable to reduce inflammation-driven emotional alterations. Supporting this assumption, SE supplementation was recently found to improve chronic LPS-induced anxiety-like behavior (60).

As anticipated, LPS-treated mice displayed 24 h post-treatment changes in the expression of many inflammatory markers, which testify to the induction of neuroinflammatory processes known to ultimately impair brain function (12, 62, 68). This implies that modulating the expression of inflammation-related genes does translate into changes in levels of corresponding proteins. Although this has not been directly demonstrated here, it has already been clearly established (69–71). In line with previous studies highlighting the immunomodulatory and anti-inflammatory properties of saffron or its bioactive components (crocins and safranal) (21, 23–26, 43, 44, 72), we showed here that the brain immune reactivity to an LPS challenge was different when mice were pretreated with SE. It notably modulated LPS-induced changes in expression of anti-inflammatory factors, as reported in the FCx and HPC, suggesting that this pretreatment likely promotes the delayed anti-inflammatory side of the response to LPS, which is set up to resolve inflammation (2, 4). Interestingly, similar modulations of anti-inflammatory gene expression recently reported following the administration of other plant extracts or nutrients, including under LPS stimulation, have been associated with changes in the polarization of macrophage/microglia from inflammatory towards anti-inflammatory states (73–76). Consistent with these findings, LPS-induced overexpression of inflammatory state markers, such as CD86 and CD11b in the STR, was blunted in mice pretreated with SE, suggesting that it may counteract the sustained microglial activation which underlies the damaging consequences of neuroinflammation on brain function (1, 2, 5, 6). Supporting this, crocins were previously shown to prevent LPS-induced increase of CD11b expression in microglial cell cultures (43, 77). SE-LPS-treated mice also exhibited dampened upregulation of the expression of IP-10, a chemokine whose modulation is indicative of that of its inducer interferon-γ (IFN-ϒ) (78). Besides, administration of saffron or crocins has been reported to reduce the induction of IFN-ϒ in different models of immune diseases (49, 79). Noteworthy, both IP-10 and IFN-ϒ have been associated with inflammation-induced KYN pathway activation and related development of neuropsychiatric symptoms (80–84). Therefore, their modulation by saffron could contribute to improve the harmful consequences of inflammation. This issue, however, needs to be investigated further, as well as the potential contribution of other inflammatory factors likely to be also targeted by saffron.

This study demonstrates for the first time that the impact of saffron on neuroinflammation extends to the downstream neurobiological systems known to trigger the neuropsychiatric comorbidities of inflammation, notably the BH4 and KYN pathways (2, 3, 5) which were activated here, as anticipated, 24 h after LPS administration. The lack of significant induction of KYN enzymes in the HPC may seem unexpected, but it actually fits with previous data reporting earlier peaks of expression, as well as different temporal expression patterns depending on the brain areas (62, 68). Besides, several studies have confirmed that the increase in levels of KYN and its derivatives measured in the FCx and STR 24 h post-LPS was also significant in the HPC (13, 63, 68, 85). Here, we showed that pretreatment with SE blunted the LPS-induced increase in expression of GTPCH1 in the STR and KYN enzymes in the FCx and STR. In line with these last results, the multivariate analysis reveals a shifted in the average score of the LPS groups pretreated with SE towards lower KYN pathway activation, as compared to those receiving only LPS. Importantly, the more contributive variables were KYN enzymes belonging to the neurotoxic branch of the pathway. Together, these findings argue for a protective role of saffron pretreatment against inflammation-driven neuronal insults, as already reported regarding stress-induced (86) or chemical treatment-induced neurotoxicity (33, 87). This notion is further supported by the reduced microglial activation also displayed by SE-LPS treated mice since the production of KYN neurotoxic derivatives occurs precisely in activated microglia (5, 9, 13). Moreover, we recently showed that SE pretreatment also reduced stress-induced KYN-related neurotoxicity in the FCx and STR (31). Interestingly, this was associated with a concomitant improvement of related depressive-like behavior, suggesting that the same could occur following the positive impact of SE on inflammation-driven KYN pathway activation. Upcoming experiments should help to test this assumption, but it already fits with recent data indicating that crocin administration ameliorates emotional behavior in different inflammatory conditions, including those related to stress or corticosterone exposure (33, 35, 88).

Compelling studies point to 5-HT and DA systems as additional targets of inflammation and important contributors of its comorbidities (1, 3, 5, 6). Consistent with this, LPS-treated mice displayed here increased 5-HIAA levels in the STR and HPC, which are indicative of enhanced 5-HT catabolism. This effect was prevented by SE pretreatment in the HPC, although it did not change the local overexpression of MAO-A induced by LPS. This might suggest that saffron counteracts the impact of LPS on 5-HT catabolism without targeting its degradation enzyme, but only the direct assessment of its enzymatic activity would allow to conclude on this issue. Besides, it should be mentioned that other mechanisms, still to be explored further, could contribute to the modulation of 5-HT system by saffron which, for example, decreases the expression of the 5-HT transporter while inflammation increases it (30, 89–91). Beyond the 5-HT system, pretreatment with SE also counteracted LPS-induced reduction in DA levels and increase in DA turnover ratios reported in the FCx. This agrees with recent studies providing evidence of a saffron-induced modulation of the DA mesocortical pathway albeit under other experimental conditions (30, 31, 33). Although the underlying mechanisms are still poorly understood, the fact that altering this pathway usually impairs behaviors related to reward processing and motivation (7, 92, 93) suggests that saffron supplementation could preferentially improve these behavioral alterations. Supporting this, crocins were recently shown to reduce anhedonia in mice (33, 88). A limitation of the current work is that it does not allow to conclude on the inflammation-induced symptoms more likely to be ameliorated by nutritional interventions with saffron or on which brain area might be most important in this regard. However, this was not the question addressed here and it would not necessarily be relevant anyway, depression being a highly multidimensional disorder that involves complex neuronal networks broadly distributed in the brain (94, 95). Instead, this study focused on the main neurobiological systems whose alteration in inflammatory conditions can contribute to the development of a large panel of behavioral symptoms. By doing this, we provided important clues to direct further experiments specifically focused on behavioral issues and thereby progress towards a better understanding of the clinical therapeutic relevance of saffron in the context of inflammation.

In conclusion, by reporting an in-depth characterization of the inflammatory and neurobiological profile displayed by saffron-pretreated mice in response to an LPS challenge, this study provides new and valuable insights on how oral administration of saffron interferes with activation of brain inflammatory processes and related changes in the activity of BH4 and KYN pathways and monoamine systems. Although more investigations are now needed, in particular to identify the behavioral implications of such modulations, including in conditions of chronic inflammation, this work reinforces the interest of investigating further the relevance of saffron-based nutritional interventions to reduce the damaging consequences of inflammation. It therefore represents an essential first step in the development of future research aiming to improve the management and treatment of inflammation-related comorbidities and to identify the clinical profile of patients likely to benefit from nutritional interventions in that context.

## Data availability statement

The raw data supporting the conclusions of this article will be made available by the authors, without undue reservation.

## Ethics statement

The animal study was approved by Comité d’éthique en expérimentation animale de l’université de Bordeaux; Plateforme APAFIS (demande d’autorisation de projets d’utilisation des animaux à des fins scientifiques). The study was conducted in accordance with the local legislation and institutional requirements.

## Author contributions

CMdO: Formal analysis, Investigation, Methodology, Writing – original draft, Writing – review & editing. JM: Investigation, Writing – review & editing. AG: Investigation, Writing – review & editing. CA: Formal analysis, Writing – review & editing. SV: Investigation, Writing – review & editing. DG: Conceptualization, Funding acquisition, Writing – review & editing. LC: Conceptualization, Funding acquisition, Writing – review & editing. LP: Methodology, Writing – review & editing. NC: Conceptualization, Funding acquisition, Investigation, Methodology, Project administration, Supervision, Writing – original draft, Writing – review & editing.

## Figures and Tables

**Table 1:** 

KYN	Kynurenine
BH4	Tetrahydrobiopterin
IDO	Indoleamine 2,3-dioxygenase
5-HT	Serotonin
LPS	Lipopolysaccharide
DA	Dopamine
GTPCH1	GTP-cyclohydroxylase-1
SE	Saffron extract
FCx	Frontal cortex
STR	Striatum
HPC	Hippocampus
5-HIAA	5-hydroxyindoleacetic acid
DOPAC	Dihydroxyphenylacetic acid
HVA	Homovanillic acid
PCA	Principal Components Analysis
PC	Principal Component
IL-1β	Interleukin-1β
TNF-α	Tumor Necrosis Factor-α
IP-10	Interferon gamma-induced Protein 10
CCL2	CC motif Chemokine Ligand 2
IL-6	Interleukin-6
CD86/74/11b/14/36/206	Clusters of Differentiation
TLR4	Toll-Like Receptor 4
CX3CL1	CX3C motif Chemokine Ligand 1
CX3CR1	CX3C motif Chemokine Receptor 1
TGFβ	Transforming growth factor-β
Socs3	Suppressor of cytokine signaling 3
Arg1	Arginase type-1
KMO	Kynurenine 3-Monooxygenase
KAT	Kynurenine Aminotransferase
Kynu	Kynureninase
Haao	3-Hydroxyanthranilate 3,4-Dioxygenase
COMT	Catechol-O-methyltransferase
MAO	Monoamine Oxidase
