# Supplementary material for: Saffron extract interferes with lipopolysaccharide-induced brain activation of the kynurenine pathway and impairment of monoamine neurotransmission in mice
Source: Front Nutr. 2023 Oct 5;10:1267839. doi: 10.3389/fnut.2023.1267839 (PMC10585275; doi:10.3389/fnut.2023.1267839)
Supplement: Supplementary file 1 [file Table_1.DOCX]

Supplementary Material

Saffron extract interferes with lipopolysaccharide-induced brain activation of the kynurenine pathway and impairment of monoamine neurotransmission in mice

Camille Monchaux De Oliveira, Jennifer Morael, Alexandrine Guille, Camille Amadieu, Sylvie Vancassel, David Gaudout, Lucile Capuron, Line Pourtau, Nathalie Castanon

# Supplementary Table

**Table S1.** Classification of the genes of interest and their references according to the systems or pathways they belong to.

| **Category** | **Genes** | **Reference** |
| --- | --- | --- |
| Housekeeping gene | *Glyceraldehyde 3-phosphate dehydrogenase (GAPDH)* | Mm99999915_g1 |
| Inflammatory factors | *Interleukin 1_β_ (IL-1_β_)* | Mm00434228_m1 |
|  | *Tumor Necrosis Factor α (TNF-α)* | Mm00446190_m1 |
|  | *Interferon ϒ-inducible protein 10 (IP-10)* | Mm00445235_m1 |
|  | *CC motif Chemokine Ligand 2 (CCL2)* | Mm00441242_m1 |
|  | *Interleukin 6 (IL-6)* | Mm00446190_m1 |
|  | *Cluster of Differentiation 86 (CD86)* | Mm00444543_m1 |
|  | *Cluster of Differentiation 74 (CD74)* | Mm00658576_m1 |
|  | *Cluster of Differentiation 11b (CD11b)* | Mm01271262_m1 |
|  | *Cluster of Differentiation 14 (CD14)* | Mm00438094_g1 |
|  | *Toll-Like Receptor 4 (TLR4)* | Mm00445274_m1 |
|  | *CX3C motif Chemokine Ligand 1 (CX3CL1)* | Mm00436454_m1 |
|  | *CX3C motif Chemokine Receptor 1 (CX3CR1)* | Mm00438354_m1 |
| Anti-inflammatory factors | *Transforming growth factor beta (TGF_β_)* | Mm03024053_m1 |
|  | *Cluster of Differentiation 36 (CD36)* | Mm01135198_m1 |
|  | *Cluster of Differentiation 206 (CD206)* | Mm00485148_m1 |
|  | *Suppressor of cytokine signaling 3 (Socs3)* | Mm00545913_s1 |
|  | *Arginase type 1 (Arg1)* | Mm00475988_m1 |
| Monoamine degradation enzymes | *Monoamine Oxidase A (MAO-A)* | Mm00558004_m1 |
|  | *Monoamine Oxidase B (MAO-B)* | Mm00555412_m1 |
|  | *Catechol-O-methyltransferase (COMT)* | Mm00514377_m1 |
| Kynurenine pathway | *Indoleamine 2,3-Dioxygenase (IDO)* | Mm00492590_m1 |
|  | *Kynurenine 3-Monooxygenase (KMO)* | Mm00505511_m1 |
|  | *Kynureninase (KYNU)* | Mm00551012_m1 |
|  | *3-Hydroxyanthranilate 3,4-Dioxygenase (HAAO)* | Mm00517945_m1 |
|  | *Kynurenine Aminotransferase (KAT)* | Mm01351821_m1 |
| BH4 pathway | *GTP-Cyclohydrolase I (GTPCH1)* | Mm01322973_m1 |
